# Assessment of Acute Kidney Injury using MRI

**DOI:** 10.1002/jmri.29281

**Published:** 2024-02-09

**Authors:** Nicholas M. Selby, Susan T. Francis

**Affiliations:** ^1^ Centre for Kidney Research and Innovation, Academic Unit for Translational Medical Sciences, School of Medicine University of Nottingham Nottingham UK; ^2^ Department of Renal Medicine University Hospitals of Derby and Burton NHS Foundation Trust Derby UK; ^3^ Sir Peter Mansfield Imaging Centre, School of Physics and Astronomy University of Nottingham Nottingham UK; ^4^ NIHR Nottingham Biomedical Research Centre Nottingham University Hospitals NHS Trust and The University of Nottingham Nottingham UK

**Keywords:** acute tubular necrosis, ischemia‐reperfusion injury, magnetic resonance imaging, hypoxia, perfusion, inflammation, fibrosis

## Abstract

There has been growing interest in using quantitative magnetic resonance imaging (MRI) to describe and understand the pathophysiology of acute kidney injury (AKI). The ability to assess kidney blood flow, perfusion, oxygenation, and changes in tissue microstructure at repeated timepoints is hugely appealing, as this offers new possibilities to describe nature and severity of AKI, track the time‐course to recovery or progression to chronic kidney disease (CKD), and may ultimately provide a method to noninvasively assess response to new therapies. This could have significant clinical implications considering that AKI is common (affecting more than 13 million people globally every year), harmful (associated with short and long‐term morbidity and mortality), and currently lacks specific treatments. However, this is also a challenging area to study. After the kidney has been affected by an initial insult that leads to AKI, complex coexisting processes ensue, which may recover or can progress to CKD. There are various preclinical models of AKI (from which most of our current understanding derives), and these differ from each other but more importantly from clinical AKI. These aspects are fundamental to interpreting the results of the different AKI studies in which renal MRI has been used, which encompass different settings of AKI and a variety of MRI measures acquired at different timepoints. This review aims to provide a comprehensive description and interpretation of current studies (both preclinical and clinical) in which MRI has been used to assess AKI, and discuss future directions in the field.

**Level of Evidence:**

1

**Technical Efficacy:**

Stage 3

Acute kidney injury (AKI) is a syndrome characterized by a sudden worsening in kidney function. The global burden of AKI is widely recognized, including by the International Society of Nephrology and other professional bodies,^1^, ^2^ but the development of new diagnostics and therapeutics for patients with AKI has been frustratingly slow. Currently, the majority of patients with AKI who require imaging undergo B‐mode ultrasound that provides limited clinical information.^3^ Newer biomarkers of AKI in blood or urine have failed to translate into mainstream clinical care, and there are no specific therapies that are effective in treating AKI. As such, there has been growing interest in using magnetic resonance imaging (MRI) to describe and understand the pathophysiology of AKI.^4^, ^5^ The ability to derive quantitative parameters to assess kidney blood flow, perfusion, oxygenation, and tissue properties that change in the setting of inflammation and fibrosis means MRI is attractive, suitable for clinical translation, and has potential to improve patient care and research. MRI can also evaluate multiorgan dysfunction associated with AKI.^6^

This comprehensive review summarizes the current literature in the use of MRI in both preclinical and clinical AKI, including studies that test interventions and in which MRI has been used to assess distant organ effects of AKI. This is prefaced with brief explanations of clinical AKI and animal models of AKI that underlie interpretation of study results. The manuscript concludes with a discussion of emerging MRI techniques that may have a role in assessing AKI in the future.

## Importance of AKI

It has been estimated that each year 13.3 million people worldwide are affected by AKI of whom at least 20% will die during their acute illness.^7^, ^8^ The associated health economics are striking, with annual costs of AKI of >£1 billion in England and ~$24 billion in the United States.^9^, ^10^ Of those that survive the acute episode, ~25% will not fully recover kidney function and will develop or experience progression of chronic kidney disease (CKD) following AKI.^11^ Other long‐term adverse outcomes include cardiovascular events, hospital readmission and further episodes of AKI that have an additive effect.^11^, ^12^ A number of these long‐term outcomes are strongly associated with failure to recover kidney function by 3 months,^11^, ^13^ which suggests that improving recovery may be an important target for future interventions. Developing new noninvasive methods to assess and characterize renal recovery after AKI would address the major limitations of the current tools to assess recovery (estimated GFR, albuminuria), which are insensitive and nonspecific.

## Pathophysiology of AKI in Clinical Settings

AKI is defined as a sudden reduction in kidney function using changes in serum creatinine and urine output as per internationally accepted criteria.^14^ This definition categorizes AKI severity into three stages, which have been shown to have strong and incremental associations with clinical outcomes.^15^ Crucially, this definition means that AKI is an extremely heterogeneous syndrome with many different causes—AKI should not be regarded as a single condition. It follows that pathophysiology and clinical course of AKI may differ significantly between individual patients and clinical settings. Comprehensive descriptions of AKI in different settings are available in other review articles.^8^

While a full discussion of AKI etiology and management is not the focus of this review, an appreciation of this is important to understand both the opportunities and challenges related to the application of MRI in AKI. Opportunities may be realized as currently there are no tools for clinical use that reliably differentiate between different mechanisms of AKI, describe their time‐course or detect the processes relevant to maladaptive repair. This is particularly relevant when considering that very few kidney biopsies are performed as part of standard care. Challenges come from applying clinical MRI in the acute phase of AKI and interpreting changes in MRI measures across potentially heterogeneous patient groups; this emphasizes the importance of granular descriptions of patients in studies.

## Preclinical Models of AKI

Much of the current understanding of the pathogenesis of AKI and mechanisms of maladaptive repair come from animal (predominantly rodent) models.^16^, ^17^, ^18^ These have allowed detailed descriptions of the complex changes in cell biology that characterize AKI (Table 1). However, the techniques used to induce AKI may differ substantially from human disease; an appreciation of this helps interpret results of preclinical MRI studies.^16^, ^17^, ^18^, ^19^ Animal models do have advantages, including direct comparisons between MRI measures and histopathology that may help understand MRI findings in clinical cohorts. It is also possible to be precise in terms of timing of onset of AKI, to standardize the nature of kidney injury, and obtain multiple measures at identical timepoints during recovery/progression to CKD.

**TABLE 1:** Summary of the Major Pathophysiological Changes Occurring in AKI

Cellular‐Level Changes in AKI	Description
Tubular	Renal tubular cell injury (proximal and distal convoluted tubules) is central to the initiation phase of injury. This is characterized by sloughing of cells into the tubular lumen, effacement, and loss of brush border in proximal tubular segments, and the formation of tubular casts that together reduce tubular flow.
Interstitium	Interstitial edema due to increased vascular permeability or back‐leak of tubular filtrate.
Inflammatory cell infiltration	Tubular injury triggering release of proinflammatory cytokines and chemokines that initiate an inflammatory cascade, with leukocyte infiltration seen from 2 to 24 hours after injury.
Vascular	Endothelial cell damage, vascular congestion, and destabilization and rarefaction of peritubular capillaries lead to hypoxia, tubular cell apoptosis, and increased collagen production. Vasoconstriction leads to reduced RBF, and reduced number of mitochondria with disordered mitochondrial bioenergetics have also been described. Changes in hemodynamics may happen differentially, with the most profound changes seen around the corticomedullary junction, but with earlier restoration of blood flow in the outer cortex.
Glomerular	Glomerular changes including collapse of the glomerular tuft due to reduced perfusion.
Recovery/maladaptive repair	Depending on severity of tubular injury, nature of inflammatory response or other signaling (eg, DAMPS mediated via Toll‐like receptors, prolonged cell‐cycle arrest), normal recovery may follow over weeks 1–4 characterized by tubular cell repair, migration, apoptosis, and proliferation with an ensuing increase in blood flow and GFR; alternatively, maladaptive repair leads to activation of profibrotic pathways, collagen deposition, vascular rarefaction, and persistent reductions in GFR and blood flow.

However, preclinical models of AKI may not adequately replicate clinical AKI, hampering clinical translation and new drug development.^20^ For example, normothermic ischemia‐reperfusion injury (IRI) involves general anesthesia, laparotomy and surgical dissection of the renal artery that is then clamped for a set time (commonly 25–50 minutes) and then released. This is clearly very different from most clinical scenarios in which AKI occurs and may partly explain human biopsy studies in which the expected histopathological changes of IRI are absent.^21^ Other common animal models of AKI include folic acid injection (which causes crystals to form in tubules with resultant tubular injury), drug‐induced tubular toxicity (eg, iodinated contrast, cisplatin, gentamicin), sepsis (eg, lipopolysaccharide (LPS) injection, or caecal ligation and perforation), and renal embolization with microspheres.^22^, ^23^ These all differ from clinical AKI by varying degrees. Additionally, many animal models of AKI fail to incorporate the effects of aging and comorbidity, which are major modifying factors in clinical cohorts.^24^ There are also differences between the anatomy of rodent and human kidneys as well as differences in innate and adaptive immune systems that may be important to some of the putative inflammatory mechanisms of AKI. Appreciating experimental AKI models and their drawbacks is important in interpreting imaging studies in which they are used. A full description of the differences between animal models and human AKI is available elsewhere.^20^

## Overview of MRI Techniques to Assess AKI

**FIGURE 1:**
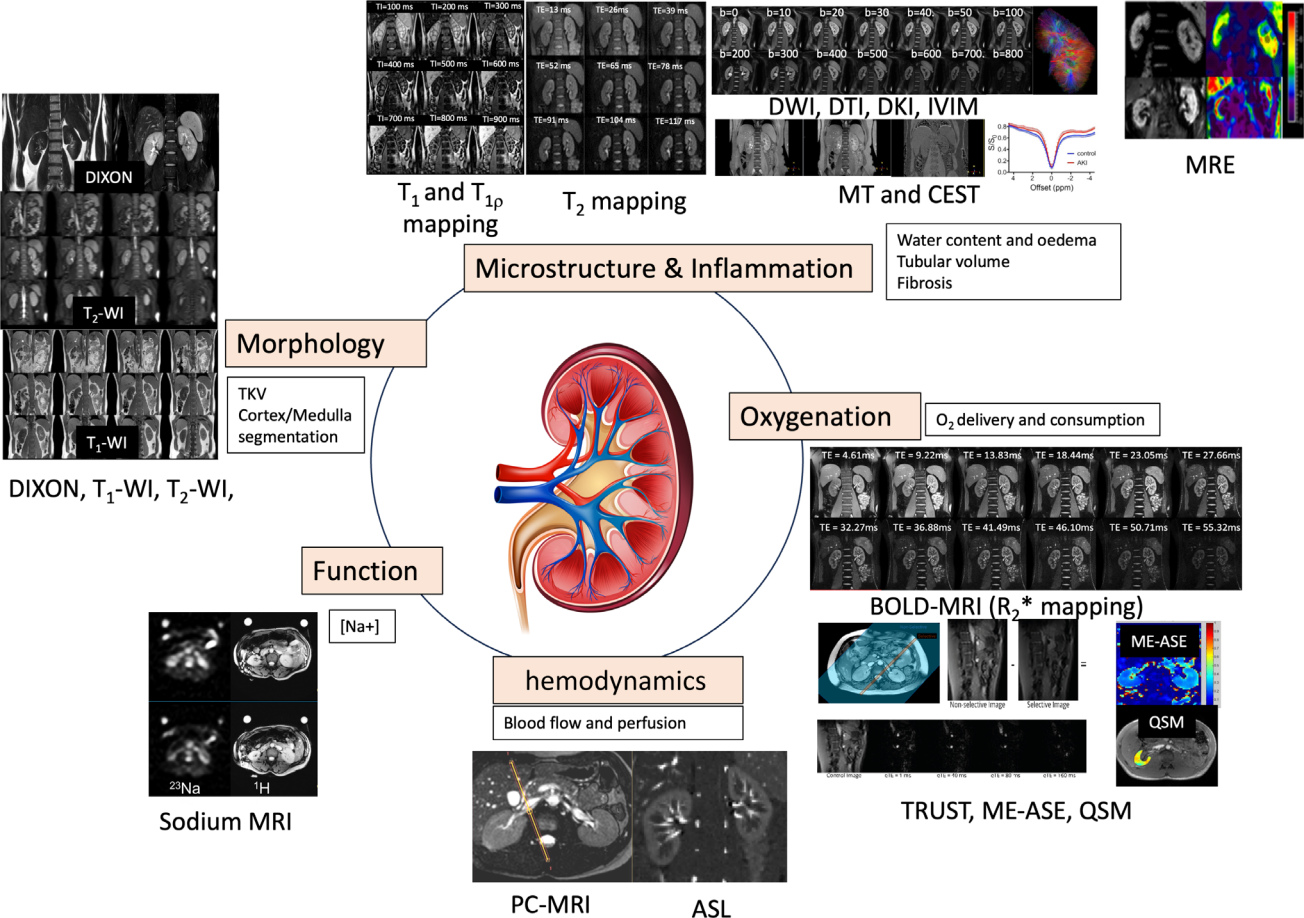
Example images of MR methods described in this review to study morphology, microstructure and inflammation, oxygenation, hemodynamics, and function. ASL = arterial spin labeling; BOLD‐MRI/*R*_2_* = blood oxygen level dependent; DWI = diffusion‐weighted imaging; DKI = diffusion kurtosis imaging; DTI = diffusion tensor imaging; IVIM = intravoxel‐incoherent motion model of diffusion; ME‐ASE = multiecho asymmetric spin echo; MRE = MR elastography; MT = magnetization transfer imaging; PC‐MRI = phase‐contrast MRI. Kidney schematic designed by brgfx/Freepik.

A range of quantitative MRI techniques can be used to assess the kidneys, including *T*_1_‐ and *T*_2_‐weighted morphometric scans to measure total kidney volume (TKV),^25^ *T*_1_ and *T*_2_ relaxometry mapping,^26^ blood oxygen level‐dependent (BOLD) imaging, diffusion‐weighted imaging (DWI),^27^, ^28^ and diffusion tensor imaging (DTI), MR angiography and phase contrast MRI (PC‐MRI),^29^ and arterial spin labeling (ASL) to measure perfusion.^30^ Emerging methods to measure tissue properties include *T*_1ρ_ or *R*_1ρ_ (the longitudinal relaxation time or rate in the rotating frame),^31^ quantitative magnetization transfer (MT),^32^ and chemical exchange saturation transfer (CEST),^33^ quantitative susceptibility mapping (QSM),^34^ MR elastography (MRE),^35^ as well as x‐nuclei measures including hyperpolarized ^13^C and ^15^N measures,^36^, ^37^ and sodium (^23^Na) imaging.^38^ Table 2 reviews these measures and their pathophysiological phenomena with example images in Fig. 1, for addition details the reader is referred to recent renal MRI review articles.^4^, ^39^ In summary, MRI has potential to identify and quantify patterns of injury, recovery and maladaptive renal repair in AKI. Unlike with biopsy, MRI can assess the renal medulla, an area that may play an important role in the pathogenesis of AKI.^40^ Together, this may help advance the understanding of AKI subgroups, objectively assess the AKI to CKD transition, and provide information about response to current and future therapies.

**TABLE 2:** Summary of the MR Techniques and the Underlying Physiologic Phenomenon the Technique Assesses

MRI Technique	MRI Measure	Physiological Process
Morphometry		
Volumetry	TKV measured from *T*_1_‐ and/or *T*_2_‐weighted images (*T*_1_‐WI and *T*_2_‐WI) or DIXON.	Kidney length and volume and their change over time due to edema or fibrosis.
Microstructure and inflammation		
DWI	Brownian motion of tissue water molecules by acquiring data at a range of *b*‐values to measure the ADC. ADC affected by tubular flow and capillary perfusion. Molecular diffusion (*D*), pseudo‐diffusion (D*), and perfusion fraction (*f*) measured with IVIM.	Microstructural changes due to renal fibrosis, cellular infiltration (inflammatory or tumorous), or edema, as well as changes in renal perfusion and in water handling in the tubular compartment.
DTI diffusion kurtosis imaging (DKI)	Assesses diffusion directionality quantified as a percentage of spatially oriented diffusion signal FA and MD. DKI parameters include mean kurtosis (*K*).	Microstructural changes that lead to a change in the preferred direction of water diffusion, for instance, tubular dilatation, tubular obstruction, or a loss in the organization of medullary tubules.
*T*_1_ mapping	Tissue‐specific relaxation time that is field strength dependent, and which can distinguish microstructural tissue composition, and can be used to assess cortico‐medullary difference (CMD).	Changes in the molecular environment. *T*_1_ is sensitive to, eg, water content, fibrosis (due to the association of collagen with supersaturated hydrogel), and inflammation (interstitial edema and cellular swelling).
*T*_2_ mapping	Tissue‐specific relaxation time that is field strength dependent that can detect changes with tissue water content, and can be used to assess CMD.	Changes in the molecular environment. *T*_2_ is sensitive to the effects of edema and/or inflammation.
MT	Dependent on the fraction of large macromolecules or immobilized cell membranes in tissue, and can be used to estimate MTR and PSR.	The fraction of large macromolecules or immobilized cell membranes in tissue; in the kidney, correlates with fibrosis.
CEST	CEST MRI results assesses the Z‐spectra, in which the normalized water signal saturation (Ssat/S0) is measured as a function of saturation frequency. Detects molecular and pH changes.	CEST is used to assess the acid–base homeostasis in the kidney and for monitoring pH that changes in several disease models.
CFE‐MRI	A superparamagnetic contrast agent for MRI. CF binds to the glomerular basement membrane due to the latters negative charge.	Provides a measure of glomerular number and size. Not for use in humans.
Hemodynamics		
PC MRI	Measure RBF in arteries and veins. Flow sensitized using bipolar gradients affecting the phase of spins that flow with a uniform velocity in the direction parallel to the gradients. Global kidney perfusion estimated by dividing total RBF to the kidney by TKV.	Increased renal resistance to flow due to downstream microvascular obstruction, large‐vessel arterial disease, or changes in systemic hemodynamics.
ASL	Subtraction technique where arterial blood water is labeled (inverted) prior to imaging. Difference signals are determined and quantified to estimate perfusion.	Cortical perfusion can be affected by a number of pathophysiological processes in acute and chronic renal disease.
Oxygenation		
BOLD	Deoxyhemoglobin is paramagnetic and shortens the transverse relaxation constant *T*_2_* (inverse of relaxation rate *R*_2_*). *R*_2_* is also influenced by changes in hematocrit and tissue water content.	Changes in renal oxygenation or changes in the microstructure of the capillary bed. Other factors such as hydration status, dietary sodium and susceptibility effects also alter *R*_2_*.
QSM	A quantitative BOLD method that uses the phase information to determine magnetic susceptibility maps of vessels and tissue.	Sensitive to changes in oxygenation, tissue microstructure and chemical composition. Decrease in susceptibility (χ) accompanies increase of diamagnetic lipids and proteins associated with inflammation and fibrosis.
TRUST	Spin tagging of blood is used to separate the signals from venous blood from surrounding tissues. This is collected at a range of *T*_2_‐weighted echo times. By measuring *R*_2_ of the renal vein oxygen consumption of the kidney can be calculated.	In contrast to BOLD, TRUST data is not influenced by edema and hematocrit. It measures oxygen consumption of the kidney which changes due to hypoxia that may occur in AKI and CKD.
Multiecho asymmetric spin echo (ME‐ASE)	Method to quantify renal oxygenation noninvasively to quantify renal oxygen extraction fraction (OEF) from degree of *R*_2_′‐weighting in an ASE sequence.	Increased OEF occurs in AKI and has been linked with the severity of ischemic AKI.
Function		
Sodium MRI	Assess sodium distribution [Na+] within the kidney to measure the corticomedullary sodium gradient.	In healthy kidneys, sodium signal intensity gradually increases from the cortex to inner medulla. Hypoxia during AKI or tubular cell injury reduce tubular sodium transport and therefore the cortico‐medullary sodium gradient. Ischemic injury also leads to inhibition of sodium/potassium adenosine triphosphatase transporter, exacerbating cellular injury.

## Preclinical Studies Using MRI to Assess AKI

**FIGURE 2:**
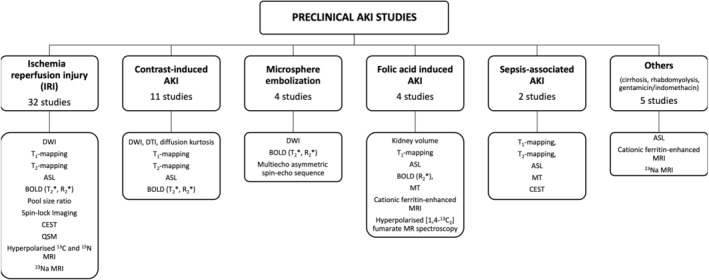
Summary of the preclinical studies using renal MRI that are included within this review, categorized by animal model of AKI and the showing the MRI measures collected across all the studies in that category.

Figure 2 provides a summary of the preclinical studies reviewed in this section, categorized by AKI model utilized and showing the range of MRI measures collected. Table 3 summarizes the findings of the studies.

**TABLE 3:** Summary of Renal MRI Changes Reported Across the Different Preclinical Models of AKI and Clinical AKI Studies

	AKI Etiology	Setting(s)	MRI Measure	Findings at Timepoints up to 7 Days	Findings at Timepoints >7 Days
Preclinical models of AKI	IRI^37^, ^38^, ^41^, ^42^, ^43^, ^44^, ^45^, ^46^, ^47^, ^48^, ^49^, ^50^, ^51^, ^52^, ^53^, ^54^, ^55^, ^56^, ^57^, ^58^, ^59^, ^60^, ^61^, ^62^, ^63^, ^64^, ^65^, ^66^, ^67^, ^68^, ^69^	Unilateral and bilateral IRI in mice (multiple strains), rats, dogs, pigs, models with contralateral nephrectomy	DWI *T*_1_‐mapping *T*_2_‐mapping ASL BOLD‐MRI MT/CEST ^23^Na MRI	↓ ADC ↑ *T*_2_ and *T*_1_ ↓ Perfusion ↑ *R*_2_* ↑ pH ↓ Corticomedullary sodium gradient and TSC	↓ADC ↑ *T*_2_ and *T*_1_ ↓ Perfusion ↑ *R*_2_* ↓ PSR, *R*_1_, *R*_2_, *R*_1r_
	Contrast‐induced AKI^70^, ^71^, ^72^, ^73^, ^74^, ^75^, ^76^, ^77^, ^78^, ^79^, ^80^, ^81^	Rats, rabbits, mice, mice/rabbit models with diabetes	DWI, DTI *T*_1_‐mapping *T*_2_‐mapping ASL BOLD‐MRI	↓ ADC, D, D*, *f* and FA ↑ *T*_1_ and *T*_2_ ↓ Perfusion ↑ *R*_2_*	↑ *T*_1_ ↓ Perfusion
	Microsphere embolization^82^, ^83^, ^84^, ^85^	Rabbits	DWI BOLD‐MRI ME‐ASE	↓ ADC Increased *R*_2_* ↑ Renal OEF	
	Folic acid induced AKI^86^, ^87^, ^88^, ^89^	Mice	Kidney volume BOLD‐MRI *T*_1_‐mapping ASL MT CFE‐MRI	↑ TKV ↑ *R*_2_* ↓ perfusion ↑ *T*_1_	↓ TKV ↑ *R*_2_* ↑ *T*_1_ ↓ Perfusion ↑ MTR ↓ Glomerular number ↑ Glomerular size
	Sepsis associated AKI, acute pancreatitis^90^, ^91^, ^92^	Mice, rats	DWI, DTI *T*_1_‐mapping *T*_2_‐mapping ASL MT	↓ ADC and FA Variable change in *T*_2_ (↑, →) ↓ Perfusion No change in *T*_1_ No change in MTR	
Clinical AKI	Varied^93^, ^94^, ^95^, ^96^, ^97^, ^98^, ^99^, ^100^, ^101^, ^102^, ^103^, ^104^	Clinical cohorts	Kidney volume *T*_1_‐mapping DWI, DTI BOLD‐MRI Phase‐contrast ASL	↑ TKV ↑ *T*_1_ Variable change in ADC (↓, →). No change in D, D*, *f* Variable change in *R*_2_* (↑, →) ↓ Renal blood flow ↓ Perfusion	↑ *T*_1_ ↓ Perfusion

DWI = diffusion weighted imaging; DTI = diffusion tensor imaging; ADC = apparent diffusion coefficient; *D* = diffusion coefficient; D* = pseudo‐diffusion coefficient; *f* = perfusion fraction; FA = fractional anisotropy; ASL = arterial spin labeling; BOLD‐MRI = blood oxygen level dependent MRI; MT = magnetization transfer; CEST = chemical exchange saturation transfer; MTR = magnetization transfer ratio; PSR = pool size ratio; TSC = total sodium concentration; ME‐ASE = multiecho asymmetric spin echo; OEF = oxygen extraction fraction; TKV = total kidney volume; CFE‐MRI = cationic ferritin enhanced MRI.

### Ischemia‐Reperfusion‐Injury

The most common preclinical model in which MRI has been applied is IRI. In a series of studies, Hueper et al measured DWI, *T*_1_‐mapping, *T*_2_‐mapping, and ASL at 7T in a unilateral IRI mouse model.^41^, ^42^, ^43^ MRI was performed at timepoints between day 1 and day 28 with comparisons of control, moderate IRI (clamp time 35 minutes) and severe IRI (clamp time 45 minutes). Following IRI, a significant increase in *T*_2_ and *T*_1_, and significant decrease in apparent diffusion coefficient (ADC) and perfusion were observed, with maximal changes seen at day 7. More severe IRI resulted in greater changes in all MRI measures, which were associated with degree of histological damage including inflammatory cell infiltration and interstitial fibrosis. MRI measures also predicted subsequent loss of kidney volume by day 28. Similar observations (fall in ADC, increased *T*_1_ and *T*_2_) have also been reported in rats with severe bilateral IRI,^44^ and reduced ADC at early timepoints following IRI in dogs.^45^ In other studies, different results were observed between mouse strains despite identical methods.^40^, ^46^, ^47^ The same group also used ASL under the same experimental set‐up to examine the effect of blood pressure agents on renal perfusion, demonstrating the ability of MRI to detect acute differences in renal perfusion between a control and hemodynamic intervention.^48^ Other groups have also demonstrated reduced renal perfusion using ASL following IRI in rats.^49^, ^50^

The effects of IRI on renal oxygenation have been assessed in studies using BOLD‐MRI. Pohlmann et al assessed *T*_2_ and *T*_2_* during the very early stages of IRI using a fast interleaved *T*_2_*/*T*_2_ mapping method at 9.4T.^51^, ^52^ *T*_2_*/*T*_2_ was performed at baseline and every 3 minutes during ischemia (45 minutes) and reperfusion (100 minutes). Changes were seen immediately after onset of the ischemia phase across all regions of the kidney. Following reperfusion, regional differences were seen comparing cortex and medulla (medullary *T*_2_*/*T*_2_ remained reduced throughout reperfusion, but cortical *T*_2_* returned to baseline and cortical *T*_2_ rose above baseline). They concluded that these results supported a role of medullary hypoxia in IRI, and the timing of changes suggested the onset of injury was immediately following reperfusion. Other studies also show greater BOLD change in the medulla. Oostendorp et al reported an expected increase in *R*_2_* (consistent with hypoxia) in the cortex and medulla of mice during ischemia.^53^ At 1‐hour after reperfusion the increased *R*_2_* persisted in the medulla, but reversed in the cortex so that values were lower than controls. This paradoxical finding either suggests increased cortical oxygenation, or the development of other factors that affect the BOLD signal. This reduction in *R*_2_* in the cortex has also been observed by others, in one study at 24 hours post IRI.^54^ Greater medullary changes (although this time without significant change in the cortical measures) were also reported by Zhang et al, measuring *R*_2_′.^55^

Wang et al studied longer‐term effects of AKI^56^ by performing multiparametric MRI at 7T on day 56 following unilateral IRI (45 minutes of ischemia). They used quantitative MT to measure pool size ratio (PSR) and MT ratio (MTR), and measured the relaxation rates of *R*_1_, *R*_2_, and *R*_2_*, as well as *R*_1ρ_ from spin‐lock imaging. Histological measures (tubular atrophy, fibrosis, and a combined score) were also collected. This model of IRI resulted in severe chronic damage with significant reduction in kidney size and large differences in tubular atrophy and fibrosis scores between ischemic and contralateral kidneys. Further, regional differences in histology were seen (more tubular atrophy in cortex and outer stripe of outer medulla (OSOM), greater fibrosis in inner stripe of the outer medulla (ISOM)). Most MRI measures were significantly different from the contralateral kidney, in particular PSR and *R*_1_ρ. Some measures (eg, *R*_2_*) were increased across all regions, but the most intriguing findings were that some measures (including PSR) changed in opposite directions when comparing the cortex and OSOM vs. ISOM. This suggests that MRI may be able to differentiate between dominant pathophysiological processes in different regions of the kidney, for example decreased PSR seen with tubular atrophy and increased apoptosis (cortex and OSOM), and increased PSR with collagen accumulation and fibrosis (ISOM). In further support of this premise but at earlier timepoints, Chen et al performed serial *T*_2_ mapping measurements with paired histology up to 48 hours following IRI.^57^ *T*_2_ was significantly increased at 1 hour after reperfusion, at which point tubular edema on histology was maximal. Importantly, at later timepoints *T*_2_ recovered in parallel with a progressive reduction in tubular edema, despite other histological changes (tubular necrosis and inflammation) becoming more severe.

Irrera et al collected CEST MRI at 7T to assess pH homeostasis and DCE‐MRI to assess perfusion in a mouse unilateral IRI model.^58^ IRI resulted in reduced perfusion, reduced filtration, and increased pH of the kidney, changes which persisted for up to 7 days. The same group performed CEST in a mouse model of unilateral IRI, comparing moderate (20 minutes) and severe (40 minutes) IRI and collecting data between days 0 and 7.^59^ Again, an increase in renal pH values were seen with changes detectable from day 1, and significant differences in degrees of change and recovery between moderate and severe IRI. There have also been individual studies of QSM^60^ and susceptibility weighted imaging^61^, ^62^ in IRI models.

A series of five studies have used hyperpolarized ^13^C and ^15^N MRI (at 3T and 9.4T) following unilateral IRI in mouse and rat models.^36^, ^37^, ^63^, ^64^, ^65^ Four studies performed MRI after 24 hours,^37^, ^63^, ^64^, ^65^ one at 7‐days,^36^ and one study was a retrospective analysis of data collected from a prior experiment.^63^ To summarize, significant changes in MRI measures were seen comparing the IRI kidney to the contralateral kidney, including signals of reduced metabolism that occurred in tandem with reduced perfusion (ASL‐MRI), and a disassociation of metabolic signals with *R*_2_* (that did correlate in the healthy kidney). Different severities of IRI (including bilateral IRI) could be distinguished with hyperpolarized MRI,^65^ and one study assessed the effects of spironolactone.^64^

^23^Na MRI has also been utilized to assess AKI. In a rat IRI model, ^23^Na MRI at 9.4T showed a reduced corticomedullary sodium gradient and reduced tissue sodium concentration (TSC) in cortex and medulla at 1 hour post‐reperfusion.^38^ Larger changes and less recovery were seen with longer ischemia times that also resulted in greater histological tubular injury. Neilsen et al reported similar patterns, albeit with a nonsignificant trend toward reduced slope of sodium from cortex to medulla.^66^ Recovery was seen at 21 days. In a study using ^23^Na MRI to examine the protective effect of contralateral nephrectomy in IRI, the corticomedullary sodium slope had a negative correlation with a urinary marker of fibrosis (type III collagen, uC3M).^54^ Rasmussen et al performed ^23^Na MRI at 3T at 1, 3, and 7 days after IRI in pigs (ischemia times of 45 minutes and 120 minutes).^67^ Results also showed a reduced corticomedullary sodium gradient as compared to control animals, alongside changes in relaxometry and diffusion measures.

Two studies have used MRI to assess therapeutic interventions to ameliorate IRI. In rats, treatment with a mitochondrial antioxidant (MitoQ) prior to IRI lessened histological damage, which was detected with *T*_2_ mapping and DCE‐MRI.^68^ Conversely, in a pig IRI model neither renal blood flow (RBF), BOLD nor ADC changed significantly in Danegaptide‐treated animals or controls (possibly suggesting IRI was too short).^69^

### Contrast‐Induced AKI (CI‐AKI)

Several studies have reported MRI measures in experimental models of CI‐AKI. However, it should be noted that these studies use contrast doses many fold‐higher than typically used in clinical practice, and the risk of CI‐AKI in clinical settings is likely much lower than much lower than previously thought.^70^

Wang et al collected *T*_1_, *T*_2_, and ASL at 3T in three groups of rats—controls (saline), CI‐AKI (iopromide, iodine dose 8 g/kg), and CI‐AKI plus a preventative treatment (Fasudil, putative anti‐inflammatory, antioxidative, and antifibrotic effects).^71^ Serial MRI measures were collected up to day 13 (quarter of the group sacrificed at each timepoint for histological analysis). Across all regions of the kidney, *T*_1_ and *T*_2_ increased in CI‐AKI group from day 1, peaked at day 3 and while *T*_1_ remained elevated, *T*_2_ recovered by day 7. Perfusion was significantly reduced from day 1, reached minimum at day 3 and was still significantly reduced by day 13. Histological findings of CI‐AKI were predominantly of tubular damage with inflammatory cell infiltration and were most severe on day 1 with only partial recovery over subsequent timepoints; an overall “histology score” prevented more granular comparisons of specific findings against MRI measures. In the Fasudil group, the histology score was less severe than the CI‐AKI group with corresponding attenuation of change in MRI measures. The same research group also reported: 1) reductions in renal diffusion and kurtosis measures with a similar temporal pattern^72^; 2) reduced intravoxel incoherent motion (IVIM) diffusion measures (pure diffusion coefficient (*D*), pseudo‐diffusion coefficient (D*), and perfusion fraction (*f*)) and increased *R*_2_* (BOLD) in normal and diabetic rabbit models of CI‐AKI (iohexol, iodine dose 2.5 g/kg), with changes persisting for longer in the diabetic group^73^; and 3) that daily administration of resveratrol for 14‐days prior to contrast administration had a protective effect with less severe reductions in *D* and *f*, and smaller increases in *R*_2_* in both cortex and outer medulla, which correlated with a lower histology score.^74^ Other studies have reported similar patterns, although with some variation in timing of peak change. In a diabetic rabbit model, DTI at 3T showed reductions in ADC and fractional anisotropy (FA) alongside an increase in *R*_2_*, all peaking at 24 hours after contrast and mostly recovering by 72 hours.^75^ Corresponding changes in hypoxia‐inducible factor‐1α expression supported that the observed changes in BOLD were caused by renal hypoxia.

In a diabetic mouse model of CI‐AKI, *D* and *f* both reduced (nadir at 24 hours), while *R*_2_* increased with early peak at 1 hour and less recovery seen in medullary regions.^76^ Zhang et al reported similar reductions in *D* (changes detectable by 12 hours, peak in cortex at 48 hours, peak in medulla at 72 hours) and in D* and *f* that were followed by recovery.^77^ Dai et al also reported similar changes in *D*, D*, and *f* in a diabetic rat model, followed by later changes in mean diffusivity (MD) and kurtosis but with all measures progressively worse until 72 hours.^78^ Tubular epithelial cell edema and tubular dilation were detectable from 1 hour onwards, inflammatory cells around tubules were seen at 48 hours and overall histology score correlated with change in MRI measures.

BOLD‐MRI was used in one study to compare routes of contrast injection, with greater increases in *R*_2_* seen with intra‐arterial contrast injection vs. intravenous administration,^79^ and DWI has been used to compare effects of low‐osmolar vs. iso‐osmolar contrast in a rat model.^80^

In addition to studies in the kidney, Yu et al demonstrated distant organ effects of CI‐AKI in the rat, showing that AKI led to changes in functional MRI of the brain (reduced amplitude of low‐frequency fluctuations) that were accompanied by behavior change and neuronal apoptosis.^81^

### Microsphere Embolization

Four studies have employed DWI as well as BOLD‐MRI and a multiecho asymmetric spin‐echo sequence to assess oxygenation to examine the effects of microsphere injection into the renal artery of rabbits, which causes vascular embolization and ischemic AKI.^82^, ^83^, ^84^, ^85^ Varying the dose of microspheres produced different severities of AKI. Together, these studies demonstrate that MRI is able to detect change very early after embolization. For example, *R*_2_* increased and ADC fell at 2 hours post embolization with severe AKI, but only changes in *R*_2_* were seen in mild AKI.^82^ At an even earlier timepoint of 1‐hour postembolization, a pulse‐shifting multiecho asymmetric spin‐echo sequence, measuring *R*_2_* and *R*_2_ simultaneously and dynamically, was able to differentiate AKI from controls whereas *R*_2_* could not.^83^ Further evidence for early renal hypoxia was provided in a study in which renal oxygenation fraction was increased at 1‐hour after embolization in both cortex and outer medulla.^84^ Kong et al compared two approaches to DWI (targeted vs. full‐field of view, using two *b*‐values, 0 and 1000 sec/mm^2^) performed immediately after embolization.^85^ Ischemic lesions (infarcts) were identified more frequently with the targeted field of view DWI, although ADC values in infarcted areas were similarly reduced with both techniques.

### Folic Acid Induced AKI

The injection of high doses of folic acid into the peritoneal cavity of rodents causes AKI (without significant toxicity in other organs, and avoiding surgery).^22^ The formation of obstructing crystals within the renal tubules leads to tubular epithelial cell injury/death, followed by proliferation, renal inflammation, and then fibrosis. The dose and injection schedule can be manipulated to create models that aim to depict AKI, the AKI‐to‐CKD transition, or CKD.

Jiang et al used multiparametric renal MRI at 16.4T to longitudinally assess mice injected with folic acid (*n* = 10) at 2 and 4 weeks, compared against control animals (*n* = 5).^86^ A comprehensive protocol of kidney volume, BOLD (*R*_2_*), *T*_1_, ASL perfusion, and MT was collected. Half of the mice were sacrificed for histology at 2 weeks, with the remainder euthanized following the 4‐week MRI scan. AKI was detectable biochemically at 2 days postinjection, and histological findings were different between 2‐ and 4‐week timepoints. At 2 weeks, tubular dilation, inflammatory infiltration, glomerular atrophy, and tubular necrosis were observed (with some degree of fibrosis on hematoxylin–eosin staining), whereas at 4 weeks tubulointerstitial atrophy and fibrosis were the predominant features. In parallel, the following changes in MRI parameters were observed. *R*_2_* increased in cortex at 2‐weeks, and in cortex and medulla by 4‐weeks. Cortical and medullary perfusion reduced at 2‐weeks with partial recovery by 4‐weeks; *T*_1_ showed reciprocal change, increasing in cortex (by 8.8%) and medulla (by 14.6%) at 2‐weeks, again with partial recovery by 4‐weeks. Kidney volume was unchanged at 2‐weeks but was ~18% smaller than control mice at 4 weeks. The MTR was unchanged at 2 weeks but was significantly increased in both cortex and medulla at 4‐weeks. MTR and *R*_2_* correlated with fibrosis in the cortex.

In another well‐conducted study, a folic acid mouse model was used to study the pathology of AKI to CKD transition using cationic ferritin‐enhanced MRI (CFE‐MRI) as well as invasive histology.^87^ Cationic ferritin was injected prior to euthanasia, which binds anionic sites in the glomeruli and shortens transverse relaxation times, with ex‐vivo imaging of the kidney allowing for whole organ assessment of glomerular/nephron number. Comparisons were made between timepoints (4 days, 4‐weeks, and 12‐weeks after folic acid injection). Key findings included an increase in kidney volume at 4 days, with subsequent loss of kidney volume at 4‐ and 12‐weeks. CFE‐MRI showed a reduction in glomerular number by 4‐weeks that was similarly reduced at 12‐weeks, with progressive compensatory increases in glomerular volume. Histological changes included dilated tubules at 4‐days, whereas collagen deposition and atubular glomeruli were seen at 4‐ and 12‐weeks. CFE‐MRI also demonstrated lower glomerular density and greater interglomerular distances (4‐ and 12‐weeks) and compared favourably to histologically‐derived metrics to discriminate early AKI (4 days) from CKD (12‐weeks). More recently, the same group used this model to identity liquid biomarkers that may be useful in assessing the AKI to CKD transition, with urinary insulin growth factor like binding protein‐3 (IGFBP‐3) and soluble tumor necrosis factor receptor II (sTNFRII) measured at time of AKI predicting structural findings on CFE‐MRI and histology at 12‐weeks.^88^

Hyperpolarized [1,4‐^13^C_2_] fumarate MR spectroscopy has also been used in a folic acid mouse model of AKI at five timepoints over the first 48 hours from injection, with the aim of detecting acute tubular necrosis (fumarate uptake increased in necrotic cells with subsequent metabolism to malate).^89^ Increased malate signal was observed at 18 hours (prior to major histological changes being visible) and this did not occur in a comparator model of glomerular disease (lupus nephritis) without tubular necrosis, showing the changes were not due to reduced kidney function or a generalized inflammatory signal. However, the malate signal was lost by 26 hours, despite abnormal renal function and severe tubular necrosis on histology that persisted to 48 hours.

### Other Experimental Models of AKI

In sepsis‐associated AKI (SA‐AKI) due to peritoneal contamination, Zhao et al collected *T*_1_, ASL‐perfusion and *T*_2_ at 18 hours in 16 rats (7 controls and 9 with sepsis).^90^ Cortical perfusion was reduced in septic animals as were cortical and medullary *T*_2_, but no differences were seen in *T*_1_. This was a difficult experiment (not all animals with sepsis sustained AKI, and there was a high mortality rate in the sepsis group), and the findings of lower *T*_2_ values suggesting reduced water content were unexpected, particularly when histology showed tubular epithelial swelling and macrophage infiltration that may be expected to result in the opposite. Lui et al studied SA‐AKI in a mouse model of intraperitoneal injection of LPS, observing that at 24 hours postinjection there were no detectable changes in *T*_1_, *T*_2_, or MTR despite increases in serum creatinine and some histological change (focal tubular luminal/brush border blurring, minimal cellular swelling, and presence of macrophages).^91^ However, changes were seen in the CEST signal, which the authors speculated could be explained by reduced blood flow or impaired mitochondrial function. In a rat model of pancreatitis that resulted in significant AKI with associated histological change, kidney diffusion measures decreased significantly from baseline by 2 hours and this persisted to study end at 8 hours.^92^

^23^Na MRI has been used in a rat model of hypoxic acute tubular necrosis induced with iodinated contrast combined with inhibition of nitric oxide and prostaglandin synthesis.^105^ The sodium gradient between cortex and outer medulla was reduced by 21%, and between cortex and inner medulla by 40%. Other experimental models in which renal MRI measures have been collected include: a rat model of cirrhosis in which reduced renal perfusion was observed^106^; glycerol‐induced rhabdomyolysis in rabbits in which renal macrophage infiltration^107^ and reduced vascular reactivity^108^ were reported; and a rabbit model of neonatal AKI induced by gentamicin and indomethacin that was used to further evaluate CFE‐MRI.^109^, ^110^

### Summary and Interpretation of Preclinical AKI Studies

**FIGURE 3:**
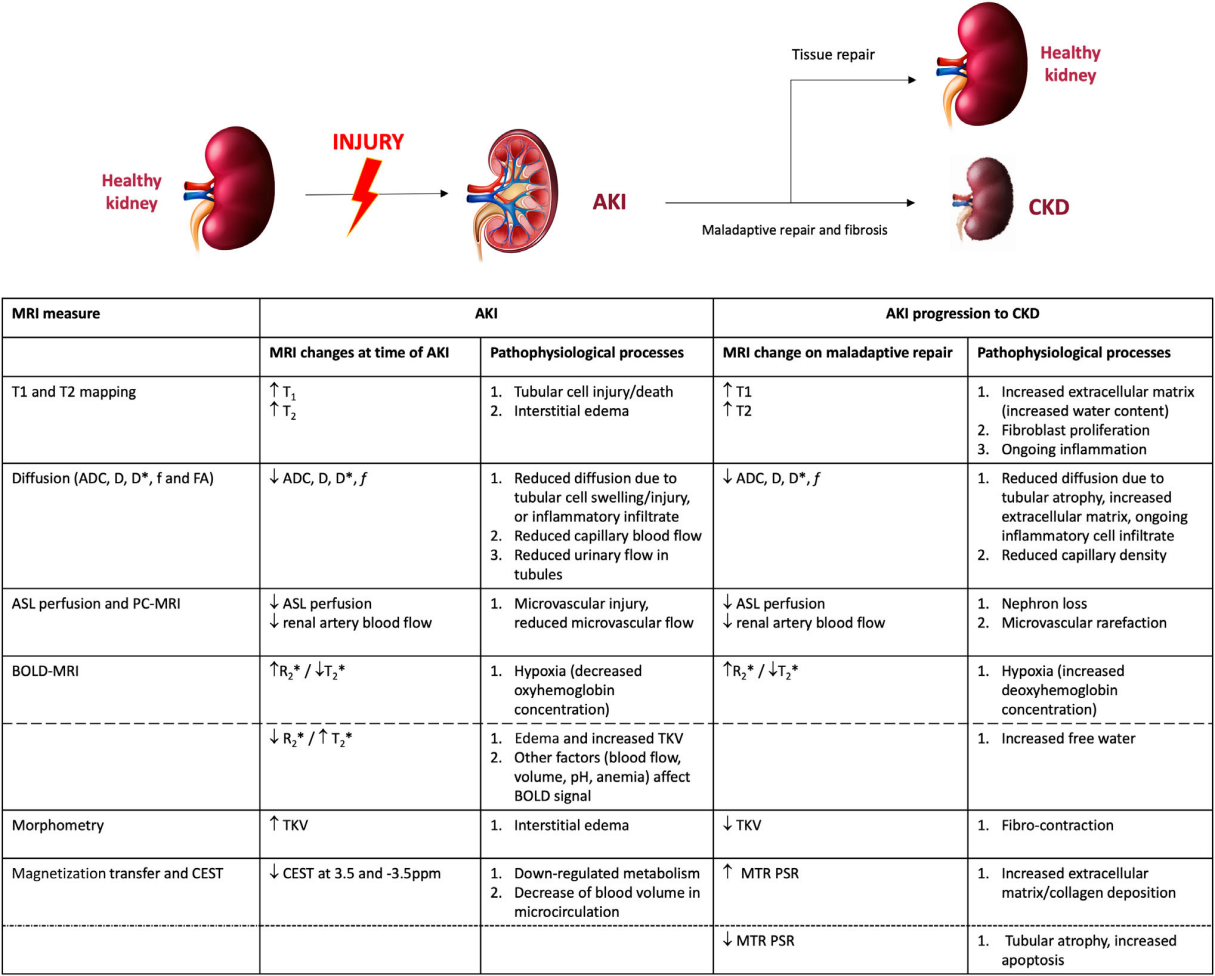
Summary of commonly reported changes in MRI measures from preclinical experimental and clinical studies at time of AKI, and then following maladaptive repair that leads to the development of CKD. For each, proposed pathophysiological changes that may underlie the changes in MRI measures are reported alongside. Many MRI measures change in the same direction at both timepoints, but are reflective of different underlying pathology (acute injury/inflammation vs. fibrosis). Exceptions to this are MTR and kidney volume. While it is not possible to make recommendations about which individual MRI measures are preferable, specific techniques may be chosen to answer research questions depending on anticipated changes in pathophysiology. Kidney schematic designed by brgfx/Freepik.

In general, animal models produce a severe AKI characterized by significant but complex pathophysiological changes, with multiple cellular, vascular, and interstitial changes occurring concurrently and often leading to chronic damage, as summarized in Table 3. It is therefore to be expected that these models result in pronounced changes in MRI measures. Changes have been reported during and after AKI in MRI measures sensitive to changes in blood flow and perfusion (ASL), oxygenation (BOLD *R*_2_*), tissue microstructure (diffusion measures, *T*_1_, *T*_2_, MTR, and QSM), water content (*T*_2_ and *T*_1_), and metabolism (CEST, hyperpolarized MRI), as well as specific techniques used to provide pathophysiological insights (eg, CFE‐MRI demonstrating reduced nephron number after AKI, or reduced cortico‐medullary sodium gradient using ^23^NaMRI suggesting reduced tubular function). Reflecting the range of processes that occur at the same time, many MRI measures change concurrently. In most studies, the severity of histological change correlated with degree of change in MRI measures, which is important to support clinical translation. This is particularly important when considering that in some studies MRI was able to discriminate subsequent progression to CKD from recovery of kidney function. However, in many studies, renal histology is not well described and it is common to see an overall score of histological damage used in analyses. This precludes more detailed understanding of which specific processes may be causing the changes in MRI measures. As shown in Fig. 3, the concurrent pathophysiological processes mean that many of the MRI measures also change together; further, some measures (eg, ASL, *T*_1_, and *R*_2_*) change at time of AKI and remain abnormal (with or without partial recovery) at later timepoints when histology has changed from a picture of tubular injury, vascular change and inflammation to one that predominantly reflects fibrosis. This may suggest that these measures are affected by more than one process (eg, *T*_1_ increasing with both tissue edema and increased extracellular matrix), or that specific pathological changes (eg, reduced blood flow/perfusion) occur across both early and later time points. There are exceptions to this, most notably MT, which did not demonstrate early change but appeared to be more specific for fibrosis at later timepoints,^56^, ^86^ and *T*_2_ mapping that may be more specific to tissue (interstitial) edema.^57^ Reduction in kidney volume was also a clear marker of chronic damage that was only detectable at later timepoints. This supports the approach of using a multiparametric protocol that uses a range of MRI measures in combination, interpreting the information that they provide individually and in combination.

There are also insights into the time course of change of MRI measures. Very early changes after renal injury could be detected by MRI in some studies—changes in the first 1–2 hours following IRI or microsphere embolization were seen predominantly in those measures sensitive to hypoxia (*R*_2_*, renal oxygenation fraction), as well as ADC (that can be affected by reduced capillary and tubular flow). Changes in medullary *R*_2_* were generally of larger magnitude and more consistently reported that those seen in the cortex (indeed, some studies reported paradoxical reductions in cortical *R*_2_* following IRI^53^, ^54^). There was some variation in time of peak/nadir change across the range of MRI measures (with possibly some of this variation arising from differences in the models of AKI), but it was interesting that some studies observed maximal change after 7 days (eg, ADC, *T*_1_‐mapping, *T*_2_‐mapping, and ASL^41^, ^42^, ^43^). It is interesting to speculate about the different pathological processes that may underlie these patterns, eg, that early tubular injury (often regarded as the initial insult in AKI) does not lead to maximal change in some MRI signals, but this occurs with the subsequent processes of interstitial edema, later tubular changes (eg, dilation and cell division) and inflammatory cell infiltration. There was some variation between studies; notably the study of AKI due to sepsis in which mild histological change was seen at 24 hours but changes in *T*_1_, *T*_2_, or MTR were not seen at that early timepoint.^91^ Repeat scans were not obtained at later timepoints, so it is not known whether these measures would have changed later.

Finally, there are examples of MRI used to assess the response to a therapeutic intervention. While the specific therapeutic interventions studied may not be immediately clinically relevant, these studies demonstrate that interventions that lessen AKI severity and ameliorate histological change can also be assessed noninvasively with MRI, which would be an additional, highly relevant application in clinical trials, particularly when considering the pipeline of new therapeutics on the horizon for AKI.

## Clinical Studies Using MRI to Assess AKI

There are fewer published MRI clinical studies in AKI compared to preclinical AKI studies. In one of the first clinical studies, Inoue et al acquired DWI and BOLD MRI measures in 23 patients with AKI (alongside 119 patients with CKD and 10 healthy volunteers (HVs)).^93^ Limited results of MRI measures in the AKI group were presented, but it was reported that neither *T*_2_* or ADC values collected at varying times within the first 10 days of hospital admission correlated with eGFR (noting that eGFR is not reliable in the setting of AKI).

Two studies have used MRI to assess critically ill patients in the intensive care unit (ICU). Prowle et al performed a free breathing PC‐MRI technique at 1.5T to measure renal artery blood flow in 10 critically ill patients with sepsis‐associated AKI and 11 HVs.^94^ Nine patients were receiving kidney replacement therapy (KRT), all had biochemical evidence of AKI stage 2 or higher and three had pre‐existing CKD. Timing of MRI scans with respect to AKI onset was not reported. RBF was significantly lower in AKI patients, and RBF as a percentage of cardiac output was consistently reduced. Recently, Luther et al used 3T renal MRI to study critically ill patients with confirmed COVID‐19, comparing 10 patients with AKI to 9 patients without AKI.^95^ The majority had AKI stage 1 (*n* = 8) with *n* = 2 having AKI stage 2/3 at the time of MRI, which was performed at a median of 1 day from AKI onset. The AKI group were more severely unwell: all of the AKI group vs. 78% of the comparator group received invasive mechanical ventilation, rates of vasopressor use between groups showed a similar pattern. Total RBF (PC‐MRI), as well as cortical and medullary perfusion (ASL) were significantly lower in the AKI group, but there were no differences in measures of oxygenation (cortical and medullary *R*_2_*, renal vein oxygen saturation) or diffusion (cortical and medullary ADC, *D*, D*, and *f*).

Further information about changes in renal perfusion were provided in a study by Dong et al who performed ASL at 3T in 11 patients with AKI and 18 HVs.^96^ Images were acquired using a pulsed ASL flow‐sensitive alternating inversion recovery sequence and single‐shot fast spin‐echo readout. Cortical perfusion was significantly reduced in the AKI group (mean 291 mL/100 g/minute, range 223–392) compared to HVs (mean 398 mL/100 g/minute, range 357–426), with a similar significant reduction in the medulla. However, these were not typical AKI patients, as they had been selected based on clinical decision to perform renal biopsy. All had intrinsic, immunological renal disease (seven had acute interstitial nephritis, two had membranous nephropathy, two had IgA nephropathy). Further, serum creatinine values in some of the AKI group were normal, casting some doubt over AKI diagnosis and potentially explaining why some patients in the AKI group had perfusion values in the HV range.

Bauer et al sought to study changes in oxygenation, and obtained BOLD‐MRI measures in a group of 9 AKI patients (one had AKI stage 1, the others had AKI stages 2/3), 10 patients undergoing nephron‐sparing nephrectomy (positive control group, as these patients had cross‐clamping of their renal artery during surgery) and 9 HVs.^97^ MRI scans were performed within 2 days (range 0–6 days) from maximum serum creatinine value. One of the strengths of this study was that six of the AKI patients underwent kidney biopsy, which showed ATN, and the other three had a clinical diagnosis of ATN confirmed by two independent nephrologists. There were no differences in mean cortical or medullary *R*_2_* values between groups, although there were three patients in the AKI group whose individual medullary *R*_2_* values were clearly higher (suggesting hypoxia) than the maximal value seen in any of the other groups; a similar pattern was seen in cortical *R*_2_* with 4 AKI patients exceeding the range of other groups. Following administration of 40 mg IV frusemide, medullary *R*_2_* fell in HVs (representing a reduction in tubular oxygenation consumption) but no significant change was seen in AKI group, or in cross‐clamped kidneys.

Derlin et al collected 1.5T renal DWI and DTI measures in 54 lung transplant patients at a mean of 14 days following surgery, along with 14 HVs.^98^ In addition to the effects of major thoracic surgery, patients were also taking immunosuppressant agents including tacrolimus (that can cause reno‐vasoconstriction) and approximately one‐fifth of patients had required postoperative extracorporeal membrane oxygenation treatment. A total of 36 patients developed AKI (17 had AKI stage 1 and 19 had AKI stage 2/3) with peak creatinine around 48 hours post‐surgery, meaning that renal function was recovering by the time of the MRI scan. Medullary ADC was significantly lower in the AKI group as compared to lung transplant patients without AKI, and was significantly higher in HVs compared to both lung transplant groups. Less marked changes were seen in cortical ADC, with the only significant difference being between the AKI group and HVs. Similar patterns suggesting greater change in the AKI group were seen in *D*, D*, and *f*. Diffusion measures correlated with eGFR on the day of the MRI scan. A different pattern was seen in DTI measures, with equal reductions in medullary FA values in all lung transplant patients regardless of AKI status, and no differences between groups in cortical FA. A complimentary study from the same group reported kidney *T*_1_ after lung and kidney transplantation, although the proportion of patients with AKI were not reported.^99^

Buchanan et al performed the only study to date that has reported MRI measures at time of AKI as well as during follow up.^100^ TKV, *T*_1_, ASL, and *R*_2_* measures were collected at 3T in nine patients at time of AKI (median of 6‐days from peak creatinine), with seven returning for follow‐up scanning at 3‐months and 1‐year. All had AKI Stage 3 of whom two required acute KRT; all had normal pre‐existing renal function. AKI was caused by sepsis in five participants, with hypovolemia (2), paracetamol overdose (1) and interstitial nephritis (1) accounting for the remainder. MRI measures at time of AKI showed increased TKV, increased cortical and medullary *T*_1_ and reduced cortical perfusion compared with the expected ranges in HVs. The degree of change was also greater than that seen in CKD. Although all patients had recovery of creatinine to baseline levels by 3‐months, complete resolution was not seen in MRI measures. TKV and *T*_1_ values decreased over time after AKI; cortical and medullary *T*_1_ (SE‐EPI) remained elevated in two patients at year 1; TKV remained above the HV range in two patients, and in another two fell to below normal into the CKD range. Cortical perfusion remained below the expected range in all but two patients by 1‐year post‐AKI. Acceptable quality BOLD *R*_2_* data were only achieved in four patients due to motion artefacts during the breath‐hold scan. Paradoxically, BOLD *R*_2_* was reduced at time of AKI (possibly relating to increased kidney volume, similar to some preclinical studies^53^, ^54^) and showed a non‐significant trend to increase over time.

The only other study to describe long‐term outcomes after AKI using MRI was performed in children, who as neonates had whole‐body hypothermia treatment for hypoxic‐ischemic encephalopathy.^101^ TKV measured by MRI was performed at 10–12 years of age in 47 participants (of whom 20 had sustained neonatal AKI, majority^16^ with AKI stage 1), alongside other measures of kidney function (cystatin‐C, iohexol GFR, and albuminuria). At long‐term follow up, only two patients had mildly reduced GFR, and one had albuminuria; there was no difference in TKV between AKI and non‐AKI groups. This may reflect the nonsevere AKI with a smaller risk of long‐term CKD that characterized the majority of patients.

Other MRI studies include a retrospective case‐series of pregnancy associated cortical necrosis, describing changes in *T*_2_‐weighted images 17–50 days postpartum,^102^ a study of patients with cirrhosis of different stages employing cardiac and renal PC‐MRI, in which a subgroup of 11 patients with AKI had the highest cardiac output and lowest RBF,^103^ and FDG PET‐MRI collected in 13 patients who sustained AKI following kidney transplantation.^104^

Distant organ effects of AKI were highlighted in a study by Isaak et al who performed cardiac MRI at a median of >2 years after an admission to ICU in 48 patients, 29 of whom had AKI requiring KRT as part of their critical illness.^111^ Despite no prior cardiac disease, ICU patients had evidence of reduced left ventricular function and greater myocardial fibrosis (*T*_1_). The AKI patients were affected more severely, with greater reductions in global longitudinal strain, a greater proportion with left ventricular segmental hypokinesia, and larger increases in myocardial *T*_1_.

### Summary and Interpretation of Clinical AKI Studies

The currently available clinical studies (summarized in Table 3) provide information on a range of AKI, including critically ill patients in ICU, groups with proven ATN, AKI from general hospital settings (in which sepsis and volume depletion are common), and a study of postoperative AKI (in the setting of lung transplantation). Studies include groups with AKI stage 1 as well as more severe AKI (stages 2/3). In studies in which >50% of participants had AKI stage 1, significant changes in MRI measures were still seen.^95^, ^98^ This highlights the sensitivity of MRI, but it is also possible that the definition of severity based on AKI stage (reflecting magnitude of change in serum creatinine) may not always indicate the degree of histopathological change. These studies also demonstrate the feasibility of obtaining renal MRI measures at time of AKI, even in critically ill patients, despite significant logistical challenges. Bearing in mind these challenges, it is surprising that there is currently only one study that performed MRI during the recovery period after AKI,^100^ when patients may find it easier to attend and comply with scan procedures (eg, breath‐holds). The potential of MRI to assess recovery after AKI was supported by observations that a significant proportion of participants had persistent abnormalities in MRI measures of perfusion, *T*_1_ and kidney volume at 1‐year despite complete biochemical recovery from AKI.^100^ This suggests that MRI may be able to detect chronic damage after an episode of AKI that cannot currently be detected in clinical practice. The findings of cardiac damage following AKI in a critical care setting suggests that using MRI to assess recovery should include multiorgan assessments. Confirmation is needed from larger studies, which should also include alternative methods of assessing renal function (eg, Cystatin C) that may be less susceptible than creatinine to over‐estimating renal function during AKI recovery.^112^

In clinical cohorts, it is much more difficult to be precise about timing of onset of renal injury, and logistical issues mean that there is variation within studies in the time after AKI that MRI scans were performed. However, it is possible to state that the majority of prior studies have performed MRI in the earlier stage of AKI (within the first 1–2 weeks), while Luther et al showed that MRI measures of blood flow and perfusion were abnormal at a median of 1 day after AKI onset (at least in the setting of critical illness).^95^ Across the studies in AKI, blood flow and perfusion were consistently reduced at time of AKI (and in some individuals also at later time points). However, there was much less consistency in BOLD measures. Indeed, several studies were unable to demonstrate differences in BOLD in patients with AKI, most notably Bauer et al in a cohort in whom ATN was clearly present.^97^ This may reflect the presence of factors other than oxygenation that influence the BOLD signal in the kidney, for example changes in renal pH, tissue edema, blood flow and blood volume, and the effects of anemia. Current results suggest that BOLD *R*_2_*/*T*_2_* measures have limitations in assessing changes in the early phases of AKI, although when BOLD was used in functional testing (with the administration of frusemide) differences between AKI and HVs were seen.^97^

Two studies did demonstrate that MRI measures sensitive to changes in tissue microstructure are altered at time of AKI, with significant increases in *T*_1_ (notably to a much greater extent than seen in CKD) coupled to increased kidney volume (together suggesting tissue edema, and/or inflammatory change),^100^ and reduced ADC with AKI following lung transplant surgery.^98^ The latter could be consistent with inflammatory infiltrate, cellular swelling or tubular injury, but the effects of reduced flow (in microcirculation and within tubules) could also be relevant.

However, it must be recognized that in addition to the relatively small number of clinical studies, the sample sizes of current studies are small. This may be a particular problem when considering the heterogeneity of AKI; individual patients may have a greater influence on results and there remain questions about generalizability. In the first instance, larger, prospective cohort studies that incorporate multiparametric MRI are required to establish renal MRI as an important tool for clinical research.

## Future Directions and Emerging MRI Techniques

Future clinical AKI studies with larger sample sizes are needed to study the pathological changes of AKI in humans, and emerging MRI techniques may provide unique opportunities in this understanding. Functional MRI measures should be used to probe the macrocirculation, microcirculation and oxygenation consumption. Prior studies suggest that even in the absence of macrovascular hemodynamic instability, microcirculatory alterations develop and play a key role in AKI. Using MRI to study both the renal macrovascular and microvascular distribution through vessel size mapping time‐of flight measures, alongside ASL perfusion and diffusion IVIM will help address this. Concurrently, *T*_1_ and *T*_2_ mapping can assess changes in renal microstructure.

Since BOLD can be complicated by edema, as well as BOLD having well‐recognized limitations of changes in *T*_2_* caused by differences in other physiological parameters such as renal blood volume fraction, tissue susceptibilities due to diamagnetic proteins, and scanner related factors such as eddy currents and magnetic field inhomogeneity, alternative measures of oxygen consumption should be considered. This includes emerging methods of quantitative oxygen delivery and consumption using *T*_2_‐relaxation‐under‐spin‐tagging (TRUST) MRI, quantitative susceptibility mapping (QSM), and asymmetric spin echo^113^ (see Table 2). Emerging MRI methods such as CEST‐based molecular imaging^91^ could be used to assess whether it is possible to measure down‐regulated cellular metabolism in AKI through detecting creatine, urea, glucose, and proteins. However for wider use of CEST MRI in the human kidney technical challenges would need to be overcome, such as those associated with the presence of fat in and around the kidney, *B*_0_ field inhomogeneity and physiological motion. Measures of tissue sodium in AKI that may detect tubular function have been studied in preclinical and pig ^23^Na MRI studies, but such methods are now feasible using sodium MRI in humans. AKI may lead to tubular injury or insufficient oxygen utilization for tubular sodium transport, manifesting as a reduction in the sodium cortico‐medullary gradient (CMG). The re‐establishment of the sodium CMG is a vital component of renal recovery and evaluation of this using ^23^Na MRI could improve classification of AKI patients' recovery.

Finally, there are a lack of clinical studies using MR to assess the degree and timings of renal recovery, and there are no clinical studies in which MRI has been used to assess response to an intervention in AKI.

## Conclusions

The evidence base to support the use of renal MRI to assess AKI has grown, particularly in preclinical studies that are significantly more numerous than clinical studies. Patterns can be seen in terms of the change in MRI measures, and identifying common themes across preclinical and clinical studies will help translation and support wider use of renal MRI in the assessment of AKI. When considering the potential of MRI to provide a step‐change in patient assessment, there is now an urgent need to build on the literature with more clinical studies, including those that assess renal recovery, and considering the inclusion of MRI in future interventional trials to assess response to therapy.
